# Efficacy and safety data in elderly patients with metastatic renal cell carcinoma included in the nivolumab Expanded Access Program (EAP) in Italy

**DOI:** 10.1371/journal.pone.0199642

**Published:** 2018-07-06

**Authors:** Maria Giuseppa Vitale, Sarah Scagliarini, Luca Galli, Sandro Pignata, Giovanni Lo Re, Alfredo Berruti, Carlotta Defferrari, Massimiliano Spada, Cristina Masini, Daniele Santini, Libero Ciuffreda, Enzo Maria Ruggeri, Carmelo Bengala, Lorenzo Livi, Daniele Fagnani, Andrea Bonetti, Lucio Giustini, Alketa Hamzaj, Giuseppe Procopio, Claudia Caserta, Roberto Sabbatini

**Affiliations:** 1 Azienda Ospedaliera Universitaria di Modena, Modena, Italy; 2 AORN A. Cardarelli, Napoli, Italy; 3 Azienda Ospedaliero Universitaria Pisana, Pisa, Italy; 4 Istituto Nazionale Tumori IRCCS Fondazione Pascale, Napoli, Italy; 5 Azienda Ospedaliera Santa Maria Degli Angeli, Pordenone, Italy; 6 Azienda Socio Sanitaria Territoriale degli Spedali Civili di Brescia, Brescia, Italy; 7 Ospedale Galliera, Genova, Italy; 8 Fondazione istituto Giglio, Cefalu, Italy; 9 Arcispedale Santa Maria Nuova di Reggio Emilia, Reggio Emilia, Italy; 10 Policlinico Universitario Campus Bio-Medico, Roma, Italy; 11 Azienda Ospedaliero Universitaria Citta della Salute e della Scienza, Torino, Italy; 12 Ospedale di Belcolle, Viterbo, Italy; 13 USL 9 Presidio Ospedaliero della Misericordia, Grosseto, Italy; 14 Università di Firenze, Firenze, Italy; 15 ASST DI Vimercate, Vimercate, Italy; 16 Mater Salutis Hospital, Legnago, Italy; 17 Area Vasta 4, Fermo, Italy; 18 Azienda USL 8 Arezzo, Italy; 19 Fondazione IRCCS Istituto Nazionale Tumori, Milano, Italy; 20 Azienda Ospedaliera Santa Maria di Terni, Terni, Italy; National Institute of Health, UNITED STATES

## Abstract

**Background:**

Results from phase III clinical trial CheckMate 025 have established nivolumab as the standard of care for treatment of metastatic renal-cell carcinoma (mRCC) after VEGF inhibitor failure; however, elderly patients are under-represented in the registration trial and little is known about the activity of nivolumab in this subgroup. The purpose of the Expanded Access Program was to provide nivolumab to patients with mRCC who had progressed despite treatment with other agents that were considered standard of care.

**Methods:**

Nivolumab 3 mg/kg was administered intravenously every 2 weeks to a maximum of 24 months or until progression or unacceptable toxicity. The current analysis included all patients from the EAP Italian cohort who had received ≥1 dose of nivolumab. Adverse events (AEs) were monitored using Common Terminology Criteria for Adverse Events v4.0.

**Results:**

A total of 389 patients with advanced RCC were enrolled in the Italian cohort of the EAP and treated with nivolumab. Of these patients, 125 (32%) were at least 70 years of age and 70 (18%) were at least 75 years of age. Efficacy with nivolumab in the elderly patients was similar to that observed in the overall EAP population and in the CheckMate 025 trial. Safety was comparable between the elderly patients and the overall EAP population, and was consistent with what previously reported.

**Conclusion:**

The final results suggest that elderly patients with pretreated metastatic RCC may benefit from therapy with nivolumab.

## Introduction

As a result of aging populations, the incidence of tumours in the elderly patients, including renal cell carcinoma (RCC), continues to increase [1].

About half of the new diagnoses of renal cell carcinoma are reported in patients over 65 years of age, particularly in 25% of cases between 65 and 74 years and in another 25% of cases over 75 years [2–3].

Nivolumab is a human immunoglobulin G4 (IgG4) monoclonal antibody (HuMAb) that selectively blocks the interaction between programmed cell death 1 (PD-1), which is expressed on activated T cells, and PD-1 ligand 1 (PD-L1) and 2 (PD-L2), which are expressed on immune cells and tumor cells. The PD-1 receptor is a negative regulator of T cell activity that has been shown to be involved in immune responses of T cells in the micro-environment tumor. The interaction of PD-1 with PD-L1 and PD-L2 ligands, expressed by the cells presenting the antigen and which can be expressed by the tumor cell or other cells in the tumor micro-environment, results in inhibition of proliferation of T cell and secretion of cytokines. Nivolumab enhances T cell responses, including anti-tumor responses, through inhibition of PD1 binding to PD-L1 and PD-L2 ligands [4–5].

The FDA approved nivolumab in November 2015 as a treatment for patients with metastatic renal cell carcinoma following prior antiangiogenic-based therapy based on the results of an open-label, randomized phase III study CheckMate-025 trial, comparing nivolumab versus everolimus.

Patients received either 3 mg/kg of IV nivolumab every 2 weeks (n = 406) or 10 mg of once daily oral everolimus (n = 397) until disease progression or unacceptable toxicity [6].

The primary endpoint was overall survival (OS) and secondary endpoints included objective response rate (ORR), progression-free survival (PFS), quality of life (QoL) and safety.

As described in detail previously [6], Nivolumab demonstrated a better ORR (26% vs 5%). No differences in terms of PFS was evident (4.2 months for nivolumab versus 4.5 months for everolimus) (hazard ratio [HR], 0.85; 95% CI, 0.73–0.99; *P* = .0371).

Nivolumab reduced the risk of death by 26% compared with everolimus in patients with previously treated mRCC, according to 3-year follow-up data from the phase III CheckMate-025 study.

Median OS was 25.8 months with nivolumab compared with 19.7 months for patients assigned to everolimus (HR, 0.74; 95% CI, 0.63–0.88; P = .0005). The 3-year OS rate with nivolumab was 39% versus 30% with everolimus.

Real-World Data from an Italian EAP confirm data from the pivotal trial and suggest that nivolumab is effective for the treatment of mRCC in routine clinical practice.

Totally, 389 pts were enrolled in the EAP across 95 Italian sites: the best overall response rate was 17% including one complete and 66 partial responses, whereas 121 (31%) had stable disease. With a median follow-up of 7 months (range, 1 to 16), 6-month and 9-month survival rates were 83% and 77%, respectively. Response and survival rates were comparable among pts regardless age, presence of brain or bone metastases and number of prior therapies [7].

The elderly represent a consistent portion of all cancer patients, but they are often under-represented in clinical trials [1].

Here, we report an analysis to evaluate the efficacy/safety profile of nivolumab in elderly (≥70 years) or very elderly (≥75 years) patients enrolled in the Italian cohort of the nivolumab Expanded Access Program.

Very elderly patients represented in the CheckMate-025 study and in the EAP, account for 8% and 19% of enrolled patients respectively.

## Materials and methods

### Patients

Eligible patients were 18 years of age or older, had histologic confirmation of advanced or metastatic renal-cell carcinoma with a clear-cell component and had received al least one prior therapy regimens (including, but not limited to, sunitinib, sorafenib, pazopanib, axitinib, tivozanib, bevacizumab, mTOR inhibitors) in the advanced or metastatic setting. Prior cytokine therapy (eg, IL-2, IFN-y), vaccine therapy, or treatment with cytotoxics was also allowed.

All patients had a Karnofsky performance status of at least 70% at the time of study enrollment.

Key exclusion criteria were central nervous system metastases (CNS), a condition requiring treatment with steroids (equivalent to >10 mg of prednisone daily), prior treatment with drugs targeting T-cell costimulation or checkpoint pathways (eg, anti-PD-1, anti-PD-L1, anti-PD-L2, anti-CD137, anti-CTLA-4), any other active malignancies or active, known, or suspected autoimmune disease or a condition requiring use of systemic immunosuppressive agents within 14 days prior to first dose. All patients signed an informed consent form.

### Study design

Nivolumab was administered at a dose of 3 mg per kilogram of body weight within 60-minute intravenous infusion every 2 weeks to a maximum of 24 months or until unacceptable toxicity, clear disease progression, or withdrawal of informed consent. Dose modifications were not permitted. Treatment beyond progression was allowed under protocol defined circumstances: investigator-assessed clinical benefit and do not have rapid disease progression, severe nivolumab-related AEs, stable performance status and no delay of imminent intervention to prevent serious complications of disease progression (eg, CNS metastases). Patient provided written informed consent prior to receiving additional nivolumab treatment. A radiographic assessment/scan was performed within 6 weeks of original PD to determine whether there has been a decrease in the tumor size or a further PD. The assessment of clinical benefit was balanced by clinical judgment as to whether the patient was clinically deteriorating and unlikely to receive any benefit from continued treatment with nivolumab.

The following medications were prohibited during the program (unless utilized to treat a drug-related adverse event): immunosuppressive agents, systemic corticosteroids > 10 mg daily prednisone equivalent and any concurrent antineoplastic therapy. Surgical resection of lesions was permitted. Supportive care for disease-related symptoms and palliative care was offered to all patients at the discretion of the treating physician. Live vaccines was avoided while on study treatment. A brief course of corticosteroids for prophylaxis (eg, for contrast dye allergy) or for treatment of non-autoimmune conditions (eg, delayed-type hypersensitivity reaction caused by a contact allergen) was permitted. Limited field palliative radiation therapy for bone pain due to pre-existing bone metastasis was permitted.

This analysis evaluated the safety and efficacy of nivolumab in patients with previously treated advanced RCC in Italian patients enrolled in a worldwide expanded access program (EAP) in elderly (≥70 years) or very elderly (≥75 years) patients enrolled in the Italian cohort of the nivolumab Expanded Access Program who had received ≥1 dose of nivolumab.

Adverse events (AEs) were monitored throughout the program and graded using the National Cancer Institute Common Terminology Criteria for Adverse Events v4.0.

Objective response rate (ORR), progression-free survival (PFS), and OS were evaluated; the method and timing of assessments were at investigator discretion.

### Study oversight

This study was approved by the institutional review board or an independent ethics committee at each center (name of the coordinator ethics committee: “Comitato Etico Regionale per la Sperimentazione Clinica della Regione Toscana”, ID number: CA209-99M_11098_201_2017) and was conducted in accordance with Good Clinical Practice guidelines, as defined by the International Conference on Harmonisation.

All the patients provided written informed consent that was based on the principles of the Declaration of Helsinki.

### Statistical analysis

Data were summarized using descriptive statistics; median and range were reported for quantitative variables and absolute frequencies and percentages for categorical items.

Survival curves were estimated with the Kaplan-Meier method.

## Results

### Patients

In total, 389 patients were enrolled in the Italian cohort of the EAP between August 2015 and April 2016 and treated with nivolumab.

Median follow-up was 11.9 (range 1–24.7). Median nivolumab dose was 13 (range, 1–49).

Of these 389 enrolled patients, 125 (32%) were at least 70 years of age and 70 (18%) were at least 75 years of age. Baseline patient characteristics are described in Table 1.

**Table 1:** Demographics and baseline patient characteristics.

Characteristics	All patients (N = 389)	≥70 years (N = 125)	≥75 years (N = 70)
**Male, n (%)**	291 (75)	94 (75)	51 (73)
**Median age (range), years**	65 (34–85)	75 (70–85)	77 (75–85)
**ECOG PS, n (%)**			
0	176 (45)	47 (38)	21 (30)
1	174 (45)	67 (54)	39 (56)
2	24 (6)	7 (6)	6 (9)
NA	15 (4)	4 (3)	4 (6)
**Metastatic site, n (%)**			
Brain	32 (8)	3 (2)	1 (1)
Bone	193 (50)	47 (38)	25 (36)
Liver	129 (33)	40 (32)	21 (30)
Lung	286 (73)	91 (73)	48 (69)
**No. of prior therapies, n (%)**			
1	80 (21)	34 (27)	21 (30)
2	137 (35)	40 (32)	25 (36)
≥3	170 (44)	50 (40)	23 (33)
NA	2 (<1)	1 (1)	1 (1)
**IMCD**			
0	62 (16)	20 (16)	12 (17)
1–2	212 (55)	72 (58)	36 (51)
> = 3	33 (8)	5 (4)	5 (7)
NA	82 (21)	28 (22)	17 (24)

NA = not available.

Median age was 65 years for all patients, and lung was the most frequent site of metastasis, 73% in all patients and patients ≥ 70 and 69% in who had ≥ 75 years. The majority of patients had an ECOG performance status 0–1 in the three subgroups of patients and had received two or more previous regimen of antiangiogenic therapy for advanced renal-cell carcinoma: 79% in all patients, 72% in patients ≥ 70 years and 69% in patients ≥ 75 years.

### Efficacy

The objective response rate was 23% for all patients, 27% for patients ≥70 years of age and 28% for patients ≥75 years of age while Stable Disease was 32%, 35% and 34% respectively.

**Fig 1:**
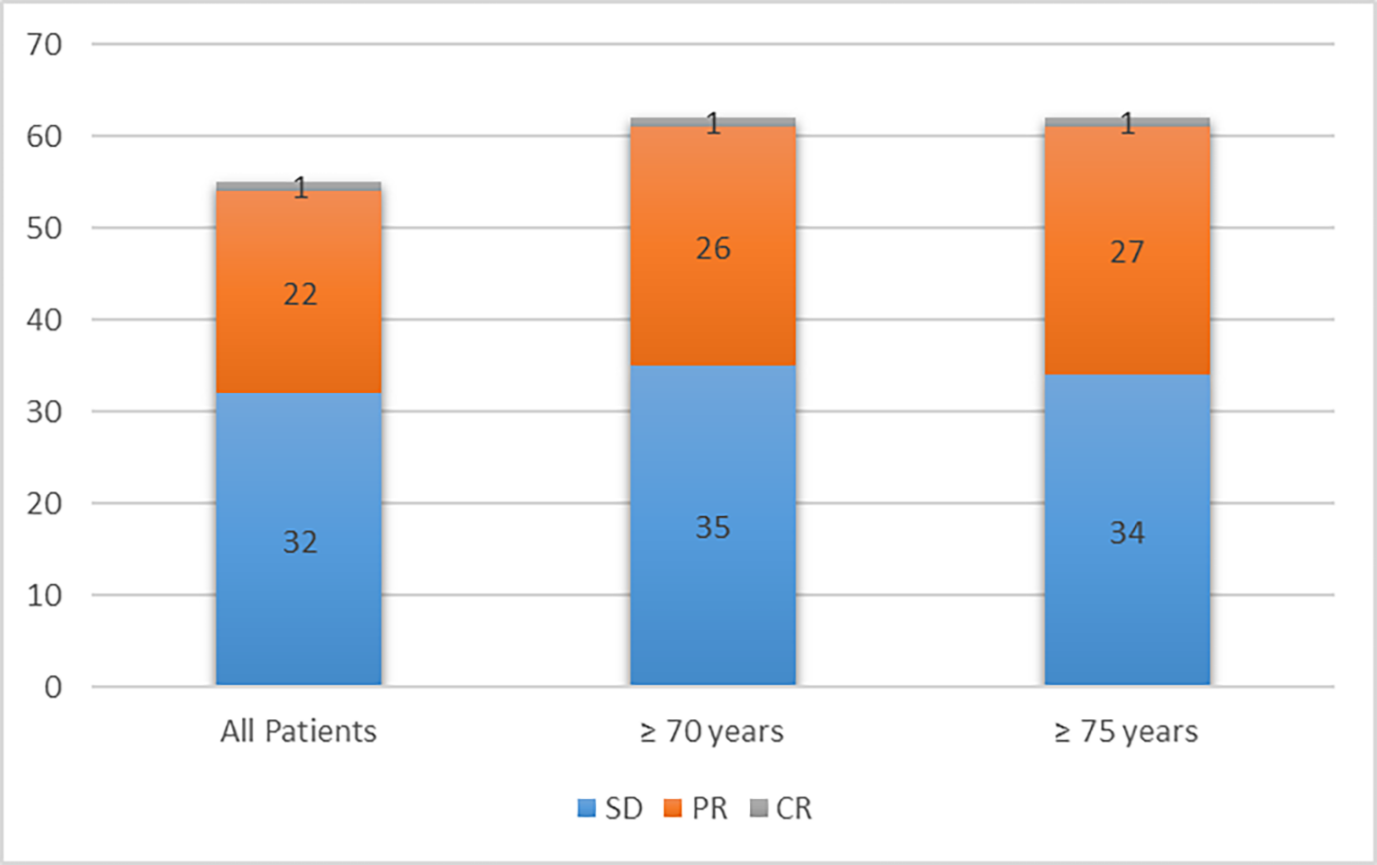
Disease control rate based on age subgroups.

Response outcomes are shown in Table 2 and Fig 1.

**Table 2:** Response outcomes based on age subgroups.

Best Response	All patients (%) N = 389	≥70 years (%) N = 125	≥75 years (%) N = 70
CR	3 (1)	1 (1)	1 (1)
PR	87 (22)	32 (26)	19 (27)
SD	124 (32)	44 (35)	24 (34)
PD	141 (36)	38 (30)	19 (27)
NA	34 (9)	10 (8)	7 (10)

CR = complete response; PD = progressive disease; PR = partial response; SD = stable disease.

The 6-, 12- and 18% month Overall Survival rates were, respectively:

- All patients = 80.2%, 64.1% and 21.8%
- ≥70 years of age = 87.2%, 77.8% and 23.2%
- ≥75 years of age = 83.6%, 77.7% and 22.8%

**Fig 2:**
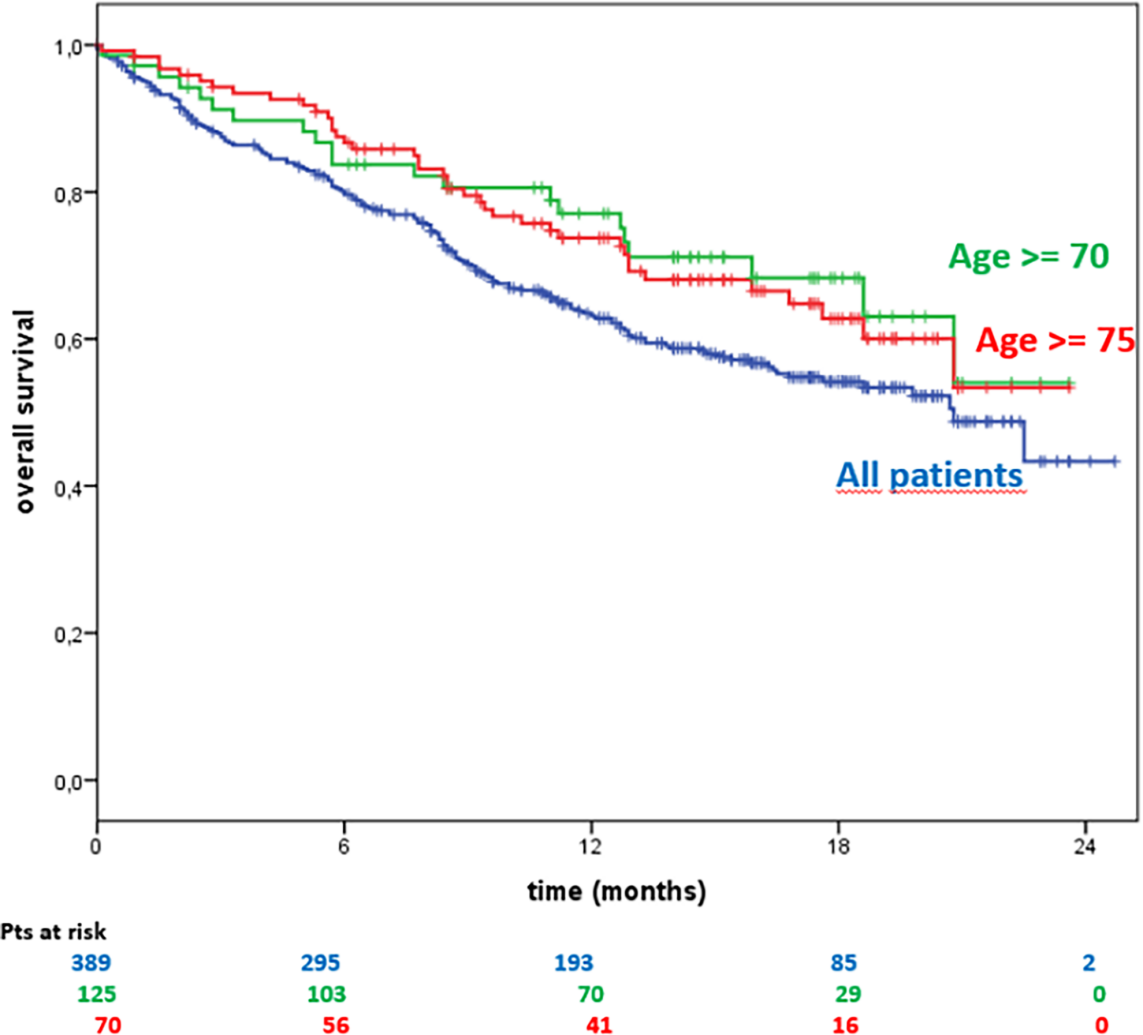
Overall survival based on age subgroups.

Kaplan–Meier estimates of OS are shown in Fig 2.

### Safety

Treatment-related adverse events of any grade occurred in 127 of the 389 patients (33%) in the general population, 37% in patients ≥70 years of age and 40% in patients ≥75 years.

Grade 3 or 4 treatment-related adverse events occurred in 27 of the 389 (7%) for all patients, in 6 of the 125 (5%) patients ≥70 years of age and in 3 of the 70 (4%) patients ≥75 years.

Although there is a slightly higher incidence of toxicity regardless the grade in the elderly and very elderly population, G3-4 Adverse Events are lower in these subgroups of patients.

The most common treatment-related AEs, of any grade, were fatigue (13%, 17% and 19% for all patients, patients ≥70 years and patients ≥75 years, respectively), skin and mucosal toxicity (10%, 9% and 11%, respectively) and gastrointestinal toxicity (9%, 10% and 13%, respectively)

The most frequent Grade 3 or 4 treatment-related adverse event was fatigue/asthenia 2%, 3% and 1% for all patients, patients ≥70 years and patients ≥75 years, respectively.

The safety outcomes for all patients, and patients ≥70 and ≥75 years, are shown in Table 3.

**Table 3:** Treatment-related AEs in ≥1% of all patients, elderly and very elderly patients.

Treatment-related AE	All patients (n = 389) Any grade, n (%)	All patients (n = 389) Grade 3–4, n (%)	Elderly (N = 125) Any grade, n (%)	Elderly (N = 125) Grade 3–4, n (%)	Very elderly (n = 70) Any grade, n (%)	Very elderly (n = 70) Grade 3–4, n (%)
**Total**	127 (33)	27 (7)	46 (37)	6 (5)	28 (40)	3 (4)
**General**						
Fatigue/asthenia	50 (13)	9 (2)	21 (17)	4 (0)	13 (19)	1 (1)
Pyrexia	12 (3)	0 (0)	5 (4)	0 (0)	2 (3)	0 (0)
Lack of appetite/anorexia	5 (1)	1 (0)	3 (2)	1 (1)	1 (1)	1 (1)
**Skin and mucosal**	39 (10)	2 (1)	11 (9)	0 (0)	8 (11)	0 (0)
Rash	34 (9)	2 (1)	9 (7)	0 (0)	6 (9)	0 (0)
**Gastrointestinal**	34 (9)	5 (1)	13 (10)	2 (2)	9 (13)	1 (1)
Diarrhea	19 (5)	3 (1)	8 (6)	1 (1)	6 (9)	1 (1)
N/V	8 (2)	2 (1)	3 (2)	1 (1)	0 (0)	0 (0)
**Pain**	9 (2)	0 (0)	5 (4)	0 (0)	4 (6)	0 (0)
**Endocrine**	13 (3)	1 (0)	5 (4)	0 (0)	2 (3)	0 (0)
Hypothyroidism	6 (2)	0 (0)	1 (1)	0 (0)	1 (1)	0 (0)
Hyperthyroidism	7 (2)	0 (0)	4 (3)	0 (0)	1 (1)	0 (0)
Autoimmune Hypophisitis	1 (0)	1 (0)	0 (0)	0 (0)	0 (0)	0 (0)
**Respiratory/pulmonary**	10 (3)	3 (1)	3 (2)	0 (0)	3 (4)	0 (0)
Pneumonitis	6 (2)	1 (0)	1 (1)	0 (0)	1 (1)	0 (0)
**Hematologic**	11 (3)	3 (1)	2 (2)	0 (0)	1 (1)	0 (0)
Anemia	9 (2)	3 (1)	2 (2)	0 (0)	1 (1)	0 (0)
**Hepatic/pancreatic**	9 (2)	0 (0)	3 (2)	0 (0)	2 (3)	0 (0)
Increased transaminase	5 (1)		2 (2)		2 (3)	

Overall, discontinuations of treatment with nivolumab in elderly and very elderly patients were consistent with those observed in the overall population (70%, 71% and 72%, respectively). Discontinuations due to treatment-related adverse events were similar in elderly patients (8%) and the overall population (8%) and slightly more frequent in very elderly patients (12%) (Table 4).

**Table 4:** Summary of discontinuations in all patients, elderly and very elderly.

Discontinuations	Patients, n (%) N = 389	Elderly Patients, n (%) N = 125	Patients, n (%) N = 70
**Discontinued treatment**	279 (72)	87 (70)	50 (71)
**Reason for discontinuation**			
PD	213 (76)	65 (75)	37 (74)
Death	21 (8)	5 (6)	2 (4)
AEs/serious AEs	22 (8)	7 (8)	6 (12)
Other	23 (8)	10 (11)	5 (10)

## Discussion

The population of elderly patients in trials conducted with molecular target drugs was always poorly represented [8]. The reasons may be various: a greater risk of adverse events and therefore reduced treatment tolerance, comorbidity, and reduced performance status. The available data are derived from only partially planned analyses performed on clinical trials or extended access programs.

Specifically, based on the results of phase III clinical trials, sunitinib, sorafenib, pazopanib, axitinib and everolimus are able to significantly increase disease-free survival regardless of the patient's age and the disease status. More recently, cabozantinib and nivolumab have been approved for the treatment of metastatic renal cell carcinoma after failure of an anti-VEGFR therapy. In METEOR trial [9], cabozantinib is able to achieve an improvement in OS compared to everolimus even in patients aged ≥65 years, while the benefit of nivolumab appears evident only in patients between 65 and 75 years (based on the results of CheckMate 025 [6]).

Some toxicities such as fatigue, anemia, appetite loss, dehydration, hand-foot-syndrome, stomatitis, diarrhoea, metabolic syndrome, infections, may contraindicate systemic treatment in elderly considered fragile patients.

The analysis of Italian expanded-access program of nivolumab in mRCC confirms the efficacy and safety of this agent in 389 patients in a real-world setting. Although the results presented here are limited by the nature of the expanded-access study design, these findings are particularly valuable due to the unselected patient population. Given that the proportion of the very elderly patients in this EAP was larger than that reported in the phase III CheckMate 025 trial (19% vs 8%), these results may provide insight into the use of nivolumab in the elderly population.

The nivolumab safety profile described here was consistent to that observed in the overall EAP population and in the CheckMate 025 trial^6^. Treatment-related AEs of any grade occurred in 33% of global population, 37% in patients ≥70 years of age and 40% in patients ≥75 years of age. These adverse events were lower compared with 79% in the CheckMate 025 trial.

Efficacy outcomes were consistent with previously published findings from selected population of CheckMate 025. The ORR was 26% in CheckMate 025 trial, 23% in EAP, 27% in EAP ≥70 years and 28% in EAP ≥75 years patients. In addition, disease control in the elderly population was 58% in over 70 years and 60% in over 75 years, compared to a disease control of 59% of CheckMate 025 population.

The tolerability profile in the two subgroups has been shown to be consistent with that of the general population. Effectiveness has also been consistent with that of the general population of EAP and also in CheckMate 025. Although, data extracted from the extended access program are retrospective, the results obtained suggest that elderly patients may benefit from nivolumab as second or subsequent line of treatment.

The landscape for managing advanced RCC is rapidly changing, with a range of drugs now approved for the treatment of this disease. Expanded-access trials are a practical way to offer a new agent not yet approved/commercially available to unselected patient population, to trial-ineligible patients in a real-world setting.

Nowadays, no analysis has specifically investigated the clinical outcomes and safety profile of nivolumab in the elderly metastatic RCC patients.

Our report has considerably extended the knowledge of the efficacy and tolerability of nivolumab in unselected real-world setting and in particular in elderly.

## Supporting information

S1 Text RCC elderly. (PDF)
