# Supplementary material for: Efficacy and safety data in elderly patients with metastatic renal cell carcinoma included in the nivolumab Expanded Access Program (EAP) in Italy
Source: PLoS One. 2018 Jul 6;13(7):e0199642. doi: 10.1371/journal.pone.0199642 (PMC6034807; doi:10.1371/journal.pone.0199642)
Supplement: S1 Text — (PDF) [file pone.0199642.s001.pdf]

Site	CODE	non_CC	Age	previous_line	Nivo_doses	Best_resp	age_70	bone_met	brain_met	age_75	sex	PFS	PROG	OS	STATUS
79.00	1	0.00	71.00	2.00	7.00	pd	2.00	0.00	0.00	1.00	1.00	2.10	yes	7.80	died
79.00	2	0.00	75.00	3.00	26.00	pr	2.00	0.00	0.00	2.00	1.00	15.20	no	15.20	alive
79.00	3	0.00	79.00	3.00	30.00	pr	2.00	1.00	0.00	2.00	1.00	17.90	no	17.90	alive
79.00	4	0.00	77.00	3.00	5.00	na	2.00	1.00	0.00	2.00	1.00	18.60	death	18.60	died
115.00	5	0.00	75.00	5.00	20.00	sd	2.00	0.00	0.00	2.00	1.00	8.30	yes	11.20	died
122.00	6	0.00	70.00	2.00	6.00	sd	2.00	1.00	0.00	1.00	1.00	7.80	death	7.80	died
135.00	7	0.00	75.00	2.00	7.00	sd	2.00	0.00	0.00	2.00	1.00	3.30	yes	7.70	died
135.00	8	0.00	80.00	1.00	18.00	pr	2.00	1.00	0.00	2.00	1.00	13.60	yes	15.20	alive
135.00	9	0.00	72.00	1.00	4.00	pd	2.00	0.00	0.00	1.00	1.00	1.30	yes	6.00	died
166.00	10	0.00	84.00	3.00	1.00	death	2.00	1.00	0.00	2.00	2.00	0.10	death	0.10	died
166.00	11	0.00	80.00	1.00	21.00	pr	2.00	0.00	0.00	2.00	1.00	10.60	no	10.60	alive
228.00	12	1.00	81.00	2.00	11.00	pd	2.00	0.00	0.00	2.00	2.00	3.40	yes	11.30	alive
228.00	13	0.00	75.00	2.00	1.00	pd	2.00	0.00	0.00	2.00	2.00	0.90	yes	0.90	lost
228.00	14	0.00	75.00	1.00	2.00	na	2.00	0.00	0.00	2.00	1.00	0.90	na	0.90	lost
228.00	15	0.00	72.00	2.00	34.00	pr	2.00	0.00	0.00	1.00	2.00	16.20	no	16.20	alive
228.00	16	0.00	79.00	2.00	4.00	pd	2.00	1.00	0.00	2.00	1.00	2.10	yes	2.20	lost
228.00	17	0.00	74.00	3.00	35.00	pr	2.00	0.00	0.00	1.00	2.00	16.10	no	16.10	alive
235.00	18	0.00	78.00	2.00	10.00	pd	2.00	1.00	0.00	2.00	1.00	2.20	yes	17.40	alive
248.00	19	0.00	74.00	2.00	6.00	pd	2.00	1.00	0.00	1.00	2.00	3.40	yes	11.30	alive
248.00	20	0.00	77.00	2.00	16.00	sd	2.00	1.00	0.00	2.00	1.00	3.60	yes	8.60	alive
248.00	21	0.00	74.00	3.00	27.00	sd	2.00	0.00	0.00	1.00	2.00	6.40	yes	20.90	alive
248.00	22	1.00	70.00	3.00	10.00	pd	2.00	0.00	0.00	1.00	1.00	3.80	yes	12.20	alive
248.00	23	0.00	75.00	1.00	25.00	pr	2.00	0.00	0.00	2.00	1.00	6.40	yes	19.00	alive
248.00	24	0.00	72.00	3.00	13.00	pd	2.00	0.00	1.00	1.00	1.00	3.70	yes	20.40	alive
248.00	25	1.00	75.00	1.00	6.00	pd	2.00	1.00	0.00	2.00	2.00	2.90	yes	14.00	alive
292.00	26	0.00	85.00	5.00	10.00	pd	2.00	0.00	0.00	2.00	1.00	5.10	yes	6.30	alive
305.00	27	0.00	71.00	4.00	29.00	sd	2.00	0.00	0.00	1.00	1.00	13.80	yes	17.60	died
315.00	28	0.00	75.00	2.00	8.00	pd	2.00	0.00	0.00	2.00	2.00	2.30	yes	5.30	died
315.00	29	0.00	78.00	2.00	10.00	pd	2.00	0.00	0.00	2.00	1.00	2.00	yes	21.00	alive
315.00	30	0.00	72.00	2.00	13.00	sd	2.00	1.00	0.00	1.00	2.00	2.00	yes	9.30	alive
321.00	31	0.00	72.00	3.00	17.00	pd	2.00	1.00	0.00	1.00	2.00	0.50	yes	9.20	alive
324.00	32	0.00	74.00	3.00	8.00	pd	2.00	1.00	0.00	1.00	1.00	2.90	yes	13.30	died

336.00	33	0.00	73.00	2.00	8.00	pd	2.00	0.00	0.00	1.00	2.00	3.10	yes	8.50	died
336.00	34	0.00	75.00	1.00	34.00	pr	2.00	1.00	0.00	2.00	1.00	17.80	no	17.80	alive
336.00	35	0.00	75.00	4.00	4.00	pd	2.00	0.00	0.00	2.00	1.00	1.70	yes	15.90	died
339.00	36	0.00	77.00	3.00	30.00	sd	2.00	0.00	0.00	2.00	1.00	4.90	yes	16.00	alive
339.00	37	0.00	70.00	5.00	19.00	sd	2.00	1.00	0.00	1.00	1.00	8.50	yes	15.20	alive
339.00	38	0.00	77.00	2.00	#NULLO!	sd	2.00	0.00	0.00	2.00	1.00	4.10	yes	6.50	lost
339.00	39	0.00	72.00	2.00	4.00	pd	2.00	1.00	0.00	1.00	1.00	2.60	yes	2.80	lost
350.00	40	0.00	81.00	#NULLO!	4.00	death	2.00	0.00	0.00	2.00	1.00	5.00	death	5.00	died
350.00	41	0.00	74.00	4.00	10.00	sd	2.00	0.00	0.00	1.00	1.00	9.60	death	9.60	died
350.00	42	0.00	70.00	3.00	26.00	sd	2.00	1.00	0.00	1.00	1.00	13.20	no	13.20	alive
379.00	43	0.00	75.00	4.00	34.00	sd	2.00	0.00	0.00	2.00	1.00	7.90	yes	22.20	alive
379.00	44	0.00	73.00	2.00	16.00	death	2.00	0.00	0.00	1.00	1.00	9.40	death	9.40	died
379.00	45	0.00	78.00	5.00	39.00	pr	2.00	0.00	0.00	2.00	1.00	19.80	no	19.80	alive
379.00	46	0.00	71.00	2.00	32.00	pd	2.00	1.00	0.00	1.00	1.00	5.90	yes	16.90	alive
379.00	47	0.00	77.00	2.00	3.00	pd	2.00	0.00	0.00	2.00	1.00	0.90	yes	2.00	died
401.00	48	1.00	83.00	3.00	10.00	sd	2.00	0.00	0.00	2.00	1.00	5.90	yes	15.20	alive
401.00	49	0.00	71.00	3.00	12.00	sd	2.00	0.00	0.00	1.00	1.00	5.50	yes	8.90	died
426.00	50	0.00	73.00	3.00	27.00	sd	2.00	1.00	0.00	1.00	1.00	15.40	no	15.40	alive
426.00	51	0.00	72.00	4.00	1.00	pd	2.00	1.00	0.00	1.00	2.00	0.00	yes	0.00	lost
426.00	52	0.00	75.00	2.00	13.00	sd	2.00	1.00	0.00	2.00	1.00	5.60	yes	14.00	alive
426.00	53	0.00	72.00	5.00	26.00	sd	2.00	0.00	0.00	1.00	2.00	12.70	no	12.70	alive
475.00	54	0.00	75.00	2.00	33.00	pr	2.00	1.00	0.00	2.00	2.00	15.90	NO	15.90	alive
534.00	55	0.00	71.00	3.00	24.00	pr	2.00	0.00	0.00	1.00	1.00	17.30	no	17.30	alive
535.00	56	0.00	76.00	1.00	14.00	pr	2.00	0.00	0.00	2.00	1.00	8.50	no	8.50	alive
568.00	57	0.00	74.00	4.00	33.00	pr	2.00	0.00	0.00	1.00	1.00	7.20	yes	20.30	alive
568.00	58	0.00	81.00	2.00	33.00	sd	2.00	0.00	0.00	2.00	2.00	3.70	yes	17.40	alive
569.00	59	0.00	82.00	1.00	15.00	sd	2.00	1.00	0.00	2.00	2.00	6.50	yes	14.10	alive
569.00	60	2.00	84.00	1.00	24.00	pr	2.00	1.00	0.00	2.00	2.00	12.40	no	12.40	alive
569.00	61	0.00	74.00	2.00	9.00	sd	2.00	0.00	0.00	1.00	1.00	7.50	yes	14.60	alive
569.00	62	0.00	81.00	1.00	27.00	pr	2.00	0.00	0.00	2.00	1.00	14.00	no	14.00	alive
601.00	63	0.00	73.00	1.00	12.00	sd	2.00	0.00	0.00	1.00	1.00	7.30	yes	9.30	died
611.00	64	0.00	70.00	3.00	7.00	pd	2.00	1.00	0.00	1.00	1.00	3.30	yes	12.20	alive
611.00	65	0.00	71.00	2.00	15.00	pr	2.00	1.00	0.00	1.00	1.00	7.30	yes	10.10	alive

615.00	66	0.00	76.00	4.00	16.00	sd	2.00	1.00	0.00	2.00	1.00	12.30	no	12.30	alive
615.00	67	0.00	81.00	1.00	2.00	na	2.00	0.00	0.00	2.00	1.00	6.10	no	6.10	lost
615.00	68	0.00	72.00	4.00	17.00	sd	2.00	1.00	0.00	1.00	1.00	8.40	no	8.40	alive
615.00	69	0.00	77.00	1.00	3.00	pd	2.00	0.00	0.00	2.00	1.00	3.20	yes	8.40	died
632.00	70	0.00	75.00	3.00	13.00	sd	2.00	0.00	0.00	2.00	1.00	10.80	no	10.80	alive
646.00	71	0.00	72.00	3.00	22.00	pr	2.00	0.00	0.00	1.00	1.00	14.00	yes	16.80	died
646.00	72	0.00	81.00	4.00	11.00	pd	2.00	0.00	0.00	2.00	1.00	2.80	yes	12.70	died
712.00	73	2.00	77.00	2.00	1.00	pd	2.00	1.00	0.00	2.00	2.00	0.50	yes	0.90	died
712.00	74	0.00	73.00	4.00	10.00	sd	2.00	1.00	0.00	1.00	1.00	6.30	yes	10.30	died
712.00	75	0.00	70.00	1.00	23.00	pd	2.00	1.00	0.00	1.00	1.00	2.50	yes	14.70	alive
731.00	76	0.00	75.00	1.00	2.00	pd	2.00	0.00	0.00	2.00	1.00	0.50	yes	1.50	died
736.00	77	0.00	78.00	2.00	30.00	sd	2.00	0.00	0.00	2.00	2.00	1.60	yes	23.60	alive
736.00	78	0.00	76.00	2.00	12.00	sd	2.00	0.00	0.00	2.00	2.00	4.40	yes	18.70	alive
736.00	79	0.00	77.00	1.00	29.00	sd	2.00	1.00	0.00	2.00	1.00	2.20	yes	18.50	alive
770.00	80	0.00	70.00	1.00	7.00	pr	2.00	0.00	0.00	1.00	1.00	6.90	no	6.90	alive
773.00	81	0.00	81.00	2.00	6.00	pd	2.00	0.00	0.00	2.00	2.00	2.10	yes	3.30	died
796.00	82	0.00	80.00	4.00	16.00	pr	2.00	1.00	0.00	2.00	1.00	8.10	yes	11.00	alive
809.00	83	0.00	71.00	5.00	5.00	sd	2.00	1.00	0.00	1.00	1.00	2.10	yes	5.30	alive
821.00	84	0.00	75.00	3.00	18.00	sd	2.00	0.00	0.00	2.00	2.00	9.20	yes	17.30	alive
841.00	85	0.00	75.00	7.00	19.00	sd	2.00	0.00	0.00	2.00	2.00	12.20	yes	14.90	alive
917.00	86	0.00	70.00	1.00	7.00	pd	2.00	0.00	0.00	1.00	1.00	2.60	yes	6.20	died
917.00	87	0.00	73.00	3.00	24.00	pr	2.00	0.00	0.00	1.00	2.00	11.60	yes	12.90	died
940.00	88	0.00	77.00	1.00	20.00	sd	2.00	1.00	1.00	2.00	1.00	10.30	yes	11.00	alive
947.00	89	0.00	77.00	2.00	31.00	cr	2.00	0.00	0.00	2.00	2.00	18.10	no	18.10	alive
1011.00	90	0.00	70.00	2.00	5.00	pd	2.00	0.00	0.00	1.00	1.00	2.70	yes	7.20	alive
1018.00	91	0.00	76.00	1.00	9.00	pr	2.00	0.00	0.00	2.00	1.00	14.40	no	14.40	alive
1018.00	92	0.00	79.00	2.00	17.00	pr	2.00	0.00	0.00	2.00	1.00	7.60	yes	12.90	died
1018.00	93	0.00	76.00	1.00	32.00	sd	2.00	0.00	0.00	2.00	1.00	14.40	no	14.40	alive
1018.00	94	0.00	73.00	1.00	3.00	pd	2.00	0.00	0.00	1.00	1.00	1.50	yes	5.60	died
1021.00	95	0.00	76.00	3.00	11.00	pd	2.00	0.00	0.00	2.00	1.00	5.90	yes	12.80	died
1047.00	96	0.00	77.00	2.00	9.00	sd	2.00	1.00	0.00	2.00	2.00	4.30	yes	22.90	alive
1047.00	97	0.00	78.00	5.00	30.00	pr	2.00	0.00	0.00	2.00	1.00	14.60	yes	14.60	alive
1047.00	98	0.00	70.00	2.00	38.00	pr	2.00	0.00	0.00	1.00	1.00	19.10	n	19.10	alive

1047.00	99	0.00	80.00	1.00	38.00	pr	2.00	0.00	0.00	2.00	1.00	19.30	n	19.30	alive
1047.00	100	0.00	70.00	2.00	5.00	pd	2.00	1.00	0.00	1.00	1.00	3.50	yes	4.20	died
1047.00	101	0.00	76.00	3.00	1.00	pr	2.00	0.00	0.00	2.00	1.00	2.80	yes	17.50	alive
1047.00	102	0.00	72.00	1.00	36.00	sd	2.00	0.00	0.00	1.00	2.00	19.40	no	19.40	alive
1047.00	103	0.00	72.00	2.00	22.00	pd	2.00	0.00	0.00	1.00	2.00	2.50	yes	18.30	alive
1047.00	104	0.00	77.00	3.00	10.00	sd	2.00	0.00	0.00	2.00	1.00	5.10	yes	11.00	died
1106.00	105	0.00	74.00	1.00	29.00	sd	2.00	0.00	0.00	1.00	1.00	17.50	yes	18.30	alive
1268.00	106	0.00	70.00	3.00	43.00	pr	2.00	1.00	0.00	1.00	1.00	0.90	yes	21.60	alive
1268.00	107	0.00	83.00	2.00	8.00	sd	2.00	1.00	0.00	2.00	1.00	2.90	yes	20.80	died
1268.00	108	0.00	77.00	2.00	43.00	pr	2.00	1.00	0.00	2.00	1.00	2.80	yes	20.90	alive
1268.00	109	0.00	70.00	1.00	33.00	sd	2.00	1.00	0.00	1.00	1.00	3.20	yes	18.70	alive
1268.00	110	1.00	71.00	3.00	4.00	pd	2.00	0.00	1.00	1.00	1.00	3.00	yes	4.90	lost
1268.00	111	0.00	78.00	2.00	3.00	death	2.00	1.00	0.00	2.00	1.00	5.70	death	5.70	died
1268.00	112	0.00	74.00	1.00	40.00	pr	2.00	1.00	0.00	1.00	1.00	19.40	no	19.40	alive
1268.00	113	0.00	75.00	1.00	34.00	sd	2.00	0.00	0.00	2.00	2.00	20.10	no	20.10	alive
1268.00	114	0.00	70.00	1.00	23.00	sd	2.00	0.00	0.00	1.00	1.00	7.40	yes	19.40	alive
1269.00	115	0.00	75.00	2.00	8.00	sd	2.00	0.00	0.00	2.00	1.00	3.70	yes	11.70	ALIVE
1269.00	116	0.00	77.00	1.00	11.00	pd	2.00	1.00	0.00	2.00	1.00	3.10	yes	5.70	died
1269.00	117	0.00	72.00	1.00	36.00	sd	2.00	1.00	0.00	1.00	1.00	12.60	yes	19.30	ALIVE
1269.00	118	0.00	74.00	1.00	38.00	pr	2.00	0.00	0.00	1.00	1.00	18.00	N	18.00	ALIVE
1269.00	119	0.00	75.00	2.00	3.00	pd	2.00	1.00	0.00	2.00	1.00	1.30	yes	2.80	died
1269.00	120	0.00	77.00	1.00	35.00	pr	2.00	0.00	0.00	2.00	1.00	18.40	N	18.40	ALIVE
1312.00	121	0.00	70.00	4.00	1.00	pd	2.00	0.00	0.00	1.00	1.00	0.00	yes	1.50	died
1320.00	122	0.00	75.00	4.00	14.00	pr	2.00	0.00	0.00	2.00	2.00	6.90	yes	20.90	alive
1320.00	123	0.00	70.00	3.00	2.00	death	2.00	0.00	0.00	1.00	1.00	5.80	death	5.80	died
1404.00	124	0.00	74.00	4.00	13.00	pr	2.00	0.00	0.00	1.00	1.00	7.10	yes	8.50	died
1502.00	125	1.00	75.00	4.00	3.00	pd	2.00	1.00	0.00	2.00	1.00	0.40	yes	2.50	died

toxicity_0_2_4	related_0_2_4	liver_met	lung_met	node_met	ecogps	G4	ORR	DCR
4.00	4.00	0.00	1.00	1.00	1.00	0.00	0.00	0.00
2.00	2.00	0.00	0.00	1.00	1.00	0.00	1.00	1.00
2.00	2.00	0.00	1.00	1.00	0.00	0.00	1.00	1.00
2.00	0.00	0.00	1.00	0.00	1.00	0.00	0.00	0.00
0.00	0.00	1.00	1.00	0.00	1.00	0.00	0.00	1.00
2.00	0.00	0.00	0.00	0.00	1.00	0.00	0.00	1.00
2.00	2.00	0.00	1.00	1.00	1.00	0.00	0.00	1.00
0.00	0.00	0.00	0.00	1.00	0.00	0.00	1.00	1.00
0.00	0.00	0.00	0.00	1.00	1.00	1.00	0.00	0.00
2.00	0.00	0.00	0.00	0.00	1.00	0.00	0.00	0.00
0.00	2.00	1.00	0.00	0.00	1.00	0.00	1.00	1.00
2.00	0.00	1.00	0.00	1.00	#NULLO!	0.00	0.00	0.00
0.00	0.00	0.00	0.00	0.00	1.00	0.00	0.00	0.00
0.00	0.00	0.00	0.00	0.00	2.00	0.00	0.00	0.00
2.00	0.00	0.00	1.00	1.00	0.00	0.00	1.00	1.00
4.00	0.00	1.00	0.00	1.00	1.00	1.00	0.00	0.00
2.00	0.00	0.00	1.00	0.00	0.00	0.00	1.00	1.00
2.00	2.00	0.00	1.00	0.00	0.00	0.00	0.00	0.00
2.00	2.00	1.00	0.00	0.00	1.00	0.00	0.00	0.00
2.00	2.00	1.00	1.00	1.00	1.00	0.00	0.00	1.00
4.00	0.00	0.00	1.00	1.00	1.00	0.00	0.00	1.00
2.00	4.00	0.00	1.00	1.00	1.00	0.00	0.00	0.00
2.00	2.00	1.00	1.00	1.00	1.00	0.00	1.00	1.00
2.00	2.00	0.00	1.00	0.00	0.00	0.00	0.00	0.00
2.00	2.00	0.00	1.00	1.00	1.00	0.00	0.00	0.00
0.00	0.00	0.00	1.00	1.00	1.00	0.00	0.00	0.00
4.00	0.00	1.00	1.00	0.00	1.00	0.00	0.00	1.00
2.00	2.00	1.00	1.00	1.00	1.00	1.00	0.00	0.00
2.00	2.00	0.00	1.00	1.00	0.00	0.00	0.00	0.00
2.00	2.00	0.00	1.00	1.00	1.00	0.00	0.00	1.00
2.00	2.00	0.00	1.00	0.00	#NULLO!	0.00	0.00	0.00
0.00	0.00	0.00	1.00	1.00	1.00	1.00	0.00	0.00

0.00	0.00	1.00	1.00	1.00	0.00	1.00	0.00	0.00
0.00	0.00	1.00	1.00	1.00	0.00	0.00	1.00	1.00
2.00	0.00	0.00	0.00	0.00	0.00	0.00	0.00	0.00
2.00	2.00	0.00	1.00	1.00	0.00	0.00	0.00	1.00
4.00	0.00	1.00	1.00	0.00	1.00	0.00	0.00	1.00
0.00	0.00	0.00	0.00	1.00	1.00	0.00	0.00	1.00
0.00	0.00	1.00	1.00	1.00	1.00	0.00	0.00	0.00
0.00	0.00	0.00	0.00	0.00	#NULLO!	0.00	0.00	0.00
0.00	0.00	0.00	1.00	1.00	1.00	0.00	0.00	1.00
0.00	0.00	1.00	0.00	0.00	0.00	0.00	0.00	1.00
4.00	4.00	1.00	1.00	0.00	1.00	0.00	0.00	1.00
0.00	0.00	0.00	1.00	0.00	0.00	0.00	0.00	0.00
2.00	0.00	1.00	1.00	0.00	1.00	0.00	1.00	1.00
2.00	0.00	1.00	1.00	1.00	1.00	0.00	0.00	0.00
2.00	0.00	0.00	1.00	0.00	2.00	0.00	0.00	0.00
0.00	0.00	0.00	0.00	1.00	0.00	0.00	0.00	1.00
2.00	0.00	0.00	1.00	0.00	1.00	0.00	0.00	1.00
0.00	0.00	0.00	0.00	0.00	1.00	0.00	0.00	1.00
0.00	0.00	0.00	1.00	0.00	1.00	0.00	0.00	0.00
0.00	0.00	0.00	1.00	1.00	0.00	0.00	0.00	1.00
0.00	0.00	0.00	1.00	1.00	1.00	0.00	0.00	1.00
0.00	0.00	0.00	1.00	1.00	2.00	0.00	1.00	1.00
2.00	2.00	0.00	1.00	1.00	0.00	0.00	1.00	1.00
0.00	0.00	0.00	1.00	1.00	0.00	0.00	1.00	1.00
2.00	2.00	1.00	0.00	0.00	1.00	0.00	1.00	1.00
2.00	2.00	0.00	1.00	0.00	1.00	0.00	0.00	1.00
2.00	2.00	0.00	1.00	0.00	1.00	0.00	0.00	1.00
2.00	2.00	0.00	1.00	0.00	1.00	0.00	1.00	1.00
0.00	2.00	1.00	1.00	1.00	0.00	0.00	0.00	1.00
0.00	0.00	0.00	1.00	0.00	0.00	0.00	1.00	1.00
2.00	0.00	0.00	1.00	1.00	0.00	0.00	0.00	1.00
0.00	0.00	1.00	0.00	1.00	1.00	0.00	0.00	0.00
0.00	0.00	0.00	1.00	0.00	0.00	1.00	1.00	1.00

0.00	0.00	0.00	1.00	1.00	1.00	0.00	0.00	1.00
2.00	2.00	0.00	1.00	1.00	1.00	0.00	0.00	0.00
2.00	2.00	0.00	1.00	0.00	0.00	0.00	0.00	1.00
2.00	0.00	0.00	1.00	1.00	1.00	0.00	0.00	0.00
0.00	0.00	0.00	1.00	1.00	0.00	0.00	0.00	1.00
2.00	2.00	1.00	1.00	1.00	1.00	0.00	1.00	1.00
0.00	0.00	1.00	0.00	1.00	1.00	0.00	0.00	0.00
0.00	0.00	0.00	1.00	1.00	2.00	0.00	0.00	0.00
2.00	2.00	0.00	1.00	1.00	0.00	0.00	0.00	1.00
0.00	0.00	0.00	0.00	0.00	1.00	0.00	0.00	0.00
0.00	0.00	1.00	1.00	0.00	2.00	1.00	0.00	0.00
2.00	2.00	1.00	1.00	0.00	1.00	0.00	0.00	1.00
4.00	4.00	1.00	0.00	0.00	2.00	0.00	0.00	1.00
0.00	0.00	0.00	1.00	0.00	1.00	0.00	0.00	1.00
2.00	0.00	0.00	1.00	0.00	0.00	0.00	1.00	1.00
2.00	2.00	1.00	1.00	1.00	1.00	1.00	0.00	0.00
4.00	4.00	0.00	1.00	0.00	1.00	0.00	1.00	1.00
2.00	0.00	0.00	1.00	1.00	0.00	0.00	0.00	1.00
0.00	0.00	0.00	1.00	0.00	0.00	0.00	0.00	1.00
0.00	0.00	0.00	1.00	0.00	0.00	0.00	0.00	1.00
0.00	0.00	0.00	1.00	1.00	0.00	0.00	0.00	0.00
2.00	0.00	0.00	1.00	0.00	0.00	0.00	1.00	1.00
0.00	0.00	0.00	1.00	1.00	#NULLO!	0.00	0.00	1.00
2.00	2.00	0.00	1.00	0.00	1.00	0.00	1.00	1.00
0.00	0.00	1.00	1.00	1.00	0.00	0.00	0.00	0.00
2.00	2.00	1.00	0.00	0.00	0.00	0.00	1.00	1.00
2.00	2.00	0.00	1.00	0.00	0.00	1.00	1.00	1.00
0.00	0.00	1.00	1.00	0.00	1.00	0.00	0.00	1.00
0.00	0.00	1.00	1.00	0.00	0.00	1.00	0.00	0.00
0.00	0.00	0.00	1.00	0.00	0.00	0.00	0.00	0.00
0.00	0.00	1.00	1.00	0.00	1.00	0.00	0.00	1.00
0.00	0.00	0.00	0.00	1.00	1.00	0.00	1.00	1.00
0.00	0.00	0.00	1.00	1.00	0.00	1.00	1.00	1.00

0.00	0.00	0.00	0.00	0.00	1.00	0.00	1.00	1.00
0.00	0.00	0.00	1.00	1.00	1.00	0.00	0.00	0.00
0.00	0.00	0.00	0.00	1.00	0.00	0.00	1.00	1.00
0.00	0.00	1.00	1.00	1.00	0.00	0.00	0.00	1.00
0.00	0.00	1.00	0.00	1.00	1.00	0.00	0.00	0.00
4.00	0.00	0.00	0.00	1.00	1.00	0.00	0.00	1.00
0.00	0.00	0.00	0.00	0.00	1.00	0.00	0.00	1.00
2.00	2.00	0.00	1.00	1.00	0.00	0.00	1.00	1.00
2.00	2.00	0.00	1.00	1.00	1.00	0.00	0.00	1.00
2.00	2.00	1.00	1.00	0.00	0.00	0.00	1.00	1.00
2.00	2.00	1.00	1.00	1.00	0.00	0.00	0.00	1.00
4.00	4.00	1.00	1.00	1.00	1.00	0.00	0.00	0.00
2.00	2.00	0.00	0.00	0.00	1.00	0.00	0.00	0.00
2.00	2.00	0.00	1.00	1.00	0.00	0.00	1.00	1.00
2.00	0.00	0.00	0.00	1.00	1.00	1.00	0.00	1.00
2.00	2.00	1.00	1.00	1.00	0.00	0.00	0.00	1.00
0.00	0.00	0.00	0.00	0.00	1.00	0.00	0.00	1.00
2.00	2.00	0.00	1.00	1.00	1.00	1.00	0.00	0.00
0.00	0.00	0.00	1.00	0.00	1.00	0.00	0.00	1.00
2.00	2.00	0.00	0.00	1.00	0.00	0.00	1.00	1.00
0.00	0.00	1.00	1.00	0.00	1.00	0.00	0.00	0.00
0.00	0.00	1.00	1.00	1.00	1.00	1.00	1.00	1.00
0.00	0.00	1.00	0.00	0.00	2.00	0.00	0.00	0.00
0.00	0.00	0.00	1.00	1.00	0.00	0.00	1.00	1.00
0.00	0.00	0.00	1.00	0.00	1.00	0.00	0.00	0.00
4.00	4.00	0.00	1.00	1.00	0.00	0.00	1.00	1.00
2.00	2.00	0.00	1.00	1.00	#NULLO!	0.00	0.00	0.00
